# Human-Specific Cortical Synaptic Connections and Their Plasticity: Is That What Makes Us Human?

**DOI:** 10.1371/journal.pbio.2001378

**Published:** 2017-01-19

**Authors:** Joana Lourenço, Alberto Bacci

**Affiliations:** Sorbonne Universités, UPMC Univ. Paris 06, CNRS UMR 7225, Inserm U1127, Institut du Cerveau et de la Moelle épinière, Paris, France

## Abstract

One outstanding difference between *Homo sapiens* and other mammals is the ability to perform highly complex cognitive tasks and behaviors, such as language, abstract thinking, and cultural diversity. How is this accomplished? According to one prominent theory, cognitive complexity is proportional to the repetition of specific computational modules over a large surface expansion of the cerebral cortex (neocortex). However, the human neocortex was shown to also possess unique features at the cellular and synaptic levels, raising the possibility that expanding the computational module is not the only mechanism underlying complex thinking. In a study published in *PLOS Biology*, Szegedi and colleagues analyzed a specific cortical circuit from live postoperative human tissue, showing that human-specific, very powerful excitatory connections between principal pyramidal neurons and inhibitory neurons are highly plastic. This suggests that exclusive plasticity of specific microcircuits might be considered among the mechanisms endowing the human neocortex with the ability to perform highly complex cognitive tasks.

The neocortex is the most evolutionarily recent region of the brain, appearing in the phylogenetic tree only in mammals. It is the most superficial structure of the mammalian brain, the final destination of all sensory information, and the site where this information is processed, stored, and used to generate complex behaviors and abstract thinking. This is accomplished by the functional interaction of six layers, each of which is specialized in different functions when processing and relaying information [1].

Importantly, diverse mammalian species perform cognitive tasks of increasing complexity differently, with *Homo sapiens* putatively being at the top of the scale. Then, what makes us human, as compared to other mammals? Is our cortex organized differently, or does it possess specific abilities in processing information?

According to one prominent hypothesis, the neocortex is organized in basic computational circuit units, which are nearly identical in all mammal species, but the number of these basic units correlates to the cognitive ability of a species. This hypothesis was put forward by V. Mountcastle, who proposed the cortical column (the ensemble of neurons encoding similar features across the whole cortical thickness) as the elementary cognitive unit, operating in parallel when present in multiple copies [2]. This idea fits well with the observation that the neocortex has an overall similar layer organization across different mammal species, but dramatically increases in surface (often resulting in complex convolutions; Fig 1A and 1B) with increased cognitive abilities of each given species. This concept justifies the use of rodents to study the basic properties of the neocortex and how the building blocks of cortical circuits lead to the emergence of some important cognitive functions. This is particularly true for mice, which can be genetically amenable and thus allow the identification and manipulation of specific elements of the cortical circuits [3].

**Fig 1:**
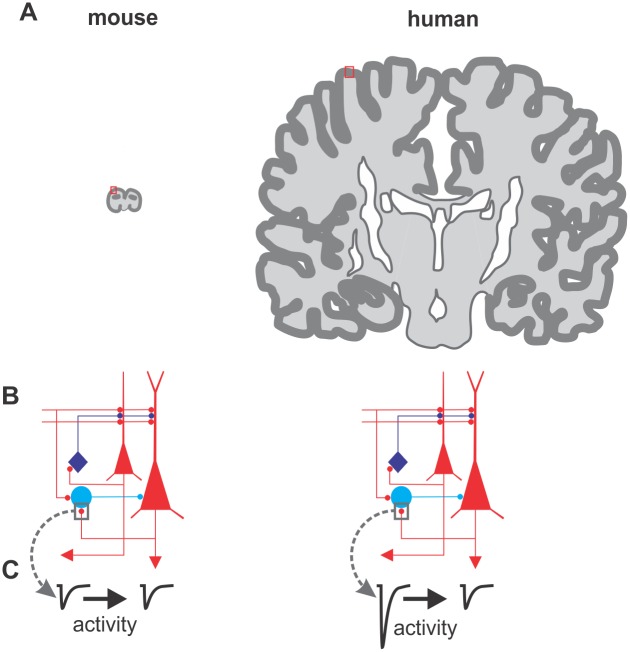
Does cortical size matter? This figure illustrates three major differences between cortices of two mammals: the mouse (widely used in neuroscience research) and *Homo sapiens*. **A:** Drawing of the cerebral hemispheres of a mouse (left) and a human (right) brain. The dark superficial region shows the neocortex, although it does not reflect actual cortical thickness. The brains are shown in the approximately same scale. Note the difference in size and convolutions between these two mammalian species, resulting in a ~1,000-fold increase in the human. This panel has been inspired by references [19–20]. **B:** Oversimplified cortical circuit diagram. In red, excitatory pyramidal neurons are shown; in blue, two general subtypes of inhibitory interneurons are shown. **C:** Despite that the two circuits look identical, they may harbor different connectivity properties: As shown by Szegedi et al, the glutamatergic connections between pyramidal neurons (PNs) and the fast-spiking (FS) interneuron (here indicated as the light-blue cell), show major functional differences: they are much larger in humans, strong enough to trigger firing of the interneuron by one PN spike. In addition, the connection shows very robust activity-dependent synaptic plasticity.

However, the size of the brain and cortex is not proportional to the actual cognitive abilities and behavioral flexibility: an elephant and a whale have a bigger brain than a primate, and, in particular, humans do not have the largest brain or cortex either in absolute or relative terms [4]. Thus, other important distinctions between humans and other mammals could derive from significant differences not only in the repetition of the basic cortical computational module but also in intrinsic features of the cortical circuit. Indeed, the human cortex can harbor specific cell types (e.g., large Betz and Meynert neurons, exclusive of primate cortices [5,6]), it may vary in neuronal density, and it may exhibit abundance of a specific cell type. Importantly, neurons that might look similar across different species have been shown to possess different physiological properties [4].

Recently, neurophysiologists started using postoperative live human tissue to characterize and analyze the physiology of cortical microcircuits. The tissue originates from patients suffering a multitude of brain diseases, ranging from brain tumors to epilepsy. Therefore, the use of these samples provided extremely useful information to study these specific disorders [7,8]. In addition, electrophysiological recordings can be done in human brain tissue not directly affected by the pathology that required the surgical treatment. This allows the investigation of human cortical circuit features in a quasi-control situation.

Using this approach, Molnar and colleagues discovered that a population of pyramidal neurons (PN) of the human neocortex makes very powerful synapses with inhibitory fast-spiking (FS) GABAergic interneurons, producing very large excitatory events in these interneurons [9] (Fig 1C). Interestingly, this feature seems to be ubiquitous across different cortical areas [10,11]. Importantly, these large PN-FS connections were not observed in nonhuman species [11], and are so large that a single spike of one pyramidal neuron can drive a postsynaptic FS cell to fire, ensuring reliable and fast feedback inhibition and initiating complex poly-synaptic cascades, affecting significant portions of the cortical neuronal network [12]. These large events are due to a presynaptic specificity, known as multi-vesicular synaptic release, which, at this synapse, is another distinct feature of human but not rodent tissue [11].

In a new study in *PLOS Biology* from K. Lamsa’s laboratory, V. Szegedi and colleagues further characterized these very strong synapses activating postsynaptic interneurons and termed them very large excitatory postsynaptic events (VLEs) [12]. Using the technically challenging electrophysiological approach of simultaneous triple patch-clamp recording, they found that two different PNs converging onto the same postsynaptic FS interneuron could elicit synaptic responses of completely different magnitude: one PN elicited VLEs, the other “normal” responses (much smaller in amplitude and comparable in size with rodents [11]). This result indicates that some PNs can recruit FS cells with an extraordinary efficacy as compared to other PNs. Importantly, these large excitatory responses are highly plastic (Fig 1C): when presynaptic PNs fired bursts of action potentials, VLEs underwent a persistent (tens of minutes) reduction in size, a synaptic plasticity phenomenon known as long-term depression (LTD). LTD of glutamatergic synapses onto interneurons have been widely described in rodents [13], but in these species, synaptic responses are normally smaller. Here Szegedi et al. found that, in humans, small-amplitude responses are poorly plastic, whereas VLEs are prone to this type of plasticity. Similarly, glutamatergic synapses between pyramidal neurons do not express VLEs or this form of plasticity [12]. The authors demonstrated that depression of large PN-FS connections relies on the activation of a specific subtype of glutamate receptor (group I metabotropic glutamate receptors) responsible for reducing glutamate release. The overall effect of LTD could be that of scaling VLEs to the size of the small responses induced by other PNs. There could be a potential relationship between multi-vesicular release [11] of VLEs and the expected consequence of high glutamate levels in and around the synapse (spillover) that might promote mGluR-dependent LTD. Indeed, mGluR-dependent LTD can be induced also in rodents, but in response to multiple-axon stimulation, which likely produces glutamate spillover. Notably, Szegedi and colleagues show that human PN-FS synapses producing VLEs can accomplish a similar job at a single-synapse level. This would result in a fine-tuned scaling capability of specific single synapses, not requiring the recruitment and synchronization of multiple axons.

As it often happens, excellent studies provide even more questions than answers. For example: Why are unitary small glutamatergic responses less susceptible to plasticity? Could they be previously depressed VLEs (and thus scaled down)? In addition, could it be that VLEs themselves had a history as weak responses and that LTD is a mechanism to restore them to normal values, a phenomenon known as metaplasticity [14]? Another intriguing possibility is that PNs producing VLEs, and therefore prone to LTD, belong to a specific subtype of neocortical principal neurons, which might be present in humans but not in rodents. Neocortical neurons can simply be divided into excitatory and inhibitory neurons. The latter are characterized by a spectacular diversity, whereas the former have been traditionally been considered more homogenous. Yet, accumulating evidence in rodents indicates that PNs can also be highly diverse, forming different subtypes according to specific morphological and functional properties, such as their target preference or common responses to specific sensory input [15]. Therefore, could a specific type of PNs (e.g., those that elicit VLEs) be what makes us human?

The VLE connections studied by Szegedi and colleagues are of particular importance. Parvalbumin (PV)-positive FS interneurons, which are often excited by VLEs, play a plethora of crucial roles within cortical circuits. Studies in rodents have identified these cells as key players for sensory information processing and for structural plasticity during the critical period [15,16]. Importantly, FS cells are specialized in inhibiting the perisomatic region of cortical PNs and control their spike timing. This generates PN synchronization and coordinated rhythmic activity, believed to underlie attention and sensory perception [17]. Recruitment of FS cells through VLEs by specific PNs might preferentially route information in the generation of neuronal assemblies, associated with network activity. In this context, activity-induced plasticity of large excitatory events onto FS interneurons adds another level of modulation of this specific cortical human microcircuit.

This study represents a technical tour de force: double and triple recordings in acute human brain slices are very challenging. Moreover, the data is hard-won, and the authors do not benefit from the mouse genetic toolbox that allows investigators to identify and/or manipulate specific cortical cell types [3]. Finally, obtaining viable postoperative tissue requires high-quality neurosurgery with additional effort, including careful handling and processing of the resected tissue material. Despite the preciousness of data from live human brain samples, there are some downsides, such as the wide age span of patients, the use of both sexes, the individuality of patients, and the fact that the tissue has to originate from diseased subjects. Moreover, the general approach of examining circuit connectivity in tissue that has been sectioned might bias the analysis of responses from specific connections. Yet, this is an issue that is unavoidable when studying the biophysics of synaptic connections and is present also in animal studies. Interestingly, however it has been recently shown that the size and short-term plasticity of unitary synaptic connections in vivo in rodents are surprisingly similar to responses obtained in resected brain slices [18]. Additionally, whereas rodents are amenable to in vivo whole-cell recordings, obviously these cannot be done in humans.

These technical weaknesses, along with the challenges inherent with neurophysiological recordings, often result in a low sample size and therefore in a reduced number of observations, as compared with animal studies. This sometimes may compromise the general interest of the findings.

Yet, results like those of Szegedi et al. are highly needed, because theirs is the only approach by which we can gain insights about specific circuit, cellular, and synaptic features of the human brain. Data obtained as in this paper can give us extremely useful information on how evolution has shaped our neocortex and thus the peculiarity of our thinking.

## Abbreviations

FS: fast-spiking
LTD: long-term depression
PNs: pyramidal neurons
VLEs: very large excitatory postsynaptic events
